# Development of nursing care guideline for burned hands

**DOI:** 10.1002/nop2.475

**Published:** 2020-03-20

**Authors:** Mojgan Lotfi, Ahmad Mirza Aghazadeh, Babak Davami, Mohammad Khajehgoodari, Hanieh Aziz karkan, Mohammad Amin Khalilzad

**Affiliations:** ^1^ Department of Medical Surgical Nursing Faculty of Nursing and Midwifery Sina Hospital Tabriz University of Medical Sciences Tabriz Iran; ^2^ Department of Basic sciences Paramedical Faculty Tabriz University of Medical Sciences Tabriz Iran; ^3^ Faculty of Medicine Sina Hospital Tabriz University of Medical Sciences Tabriz Iran; ^4^ Department of Medical Surgical Nursing Faculty of Nursing and Midwifery Tabriz University of Medical Sciences Tabriz Iran; ^5^ Laboratory Sciences Tabriz Azad University of Medical Sciences Tabriz Iran

**Keywords:** burns, hand burns, nursing guidelines, nutrition, pain control, wound management

## Abstract

**Aim:**

To develop an evidence‐based guideline to care for hand‐burned patients.

**Design:**

An integrative review.

**Method:**

The search was conducted of EMBASE, PubMed, Web of Science, SCOPUS, Clinical Key, Iranmedex, Magiran, Scientific Information Database (SID), Cochran, CINAHL and Google Scholar databases from January 2000–August 2019. Following the formation of the research team, two researchers independently selected the eligible studies. The initial search resulted in 2,230 records; ultimately, 40 articles were identified to be the review after screening the records based on the study's inclusion and exclusion criteria. Quality of selected studies was evaluated with the MMAT method.

**Results:**

Data syntheses of selected studies, coded by highlighting the relevant parts of the text, and assigning code words to these areas were done. Following this, a constant comparison was used to develop categories by combining codes. Finally, hand burns nursing care guideline was developed by categorizing descriptive themes in two main phases.

**Conclusion:**

This review results have shown that evidence‐based guidelines present high‐quality recommendations for the healthcare team, which improves the quality of clinical care. Due to a lack of established guidelines in our context, it seems to be helpful to use evidence‐based guidelines in managing burned hands.

## 1 INTRODUCTION

Burn injuries are the most common form of traumas that everyone may experience them. It is an injury to the skin or other organic tissues caused by hot liquids, hot solids, flames, radiation, radioactivity, chemicals, friction and electricity (Abu‐Sittah, El Khatib, & Dibo, 2011; Li, Deng, et al., 2017). Annually, approximately 300,000 people die due to burn injuries, of which 95% occur in low‐ and middle‐income countries. In Iran, 150,000 burns occur each year, with an annual death of 3,000 individuals (Dehghani, Hakimi, Mousazadeh, Zeynali, & Samimian, 2014; Vaghardoost, Kazemzadeh, & Rabieepoor, 2016).

Although all parts of the body are exposed to burn injury, the hands are the most frequently affected parts of the body in burn traumas (Robinson & Chhabra, 2015). According to the latest studies, the hands are affected in 80% of burn injuries due to their anatomical position in the body (Allam, Mostafa, Zayed, & El‐Gamaly, 2007; Mohaddes Ardebili, Manzari, & Bozorgnejad, 2014). Although the hands represent only 5% of total body surface area, they are the primary means by which we engage with the environmental context; therefore, hand burns may have severe functional and psychosocial implications in an individual life (Brychta, 2012; Mohaddes Ardebili et al., 2014; Robinson & Chhabra, 2015; Soni, Pham, & Ko, 2017).

By improvement in the management of patients with severe burns, including fluid resuscitation, pain management, modern dressing products, nutritional support, various surgical interventions, infection control and early rehabilitation programmes, the survival rate has been increasing in the last decades. But serious complications are still common among burn patients (Abu‐Sittah et al., 2011; Deng et al., 2016; Kamolz, Kitzinger, Karle, & Frey, 2009; Soni et al., 2017).

Due to the differences in hand burn impairment nature from other injuries, hand burns are usually associated with numerous physical and psychological problems. Deformities, disabilities, wound infection, severe pain, contractures and hypertrophic scars are the most physical difficulties that a hand‐burned survivor experiences (Abu‐Sittah et al., 2011; Afifi et al., 2016). Simultaneously with physical difficulties, hand‐burned patients confronted with multiple psychological challenges such as anxiety, post‐trauma stress disorders, depression, sleep disorders, aesthetic problems, dependency, body image dissatisfaction and low self‐esteem (Kornhaber, Wilson, Abu‐Qamar, & McLean, 2014; Titscher, Lumenta, Kamolz, Mittlboeck, & Frey, 2010).

Multiple physical and psychosocial complications following hand burn injuries emphasize the need for proper management (Bayuo, Agbenorku, & Amankwa, 2016; Luce, 2000). It has been indicated repeatedly that the use of evidence‐based guidelines by healthcare providers is a useful way of achieving optimal functional outcomes with fewer disabilities in hand burn patients (Brychta, 2012).

Clinical guidelines are specific and detailed plans that are used as a medical guide for daily clinical care. Guidelines have been designed for: (a) improving the quality of health care, (b) decreasing the use of unnecessary and ineffective procedures and (c) facilitating the patient management with high quality and fewer risk disabilities (Brychta, 2012).

According to reviews, several studies have been done in the development of clinical guidelines for the management of patients with hand burns; however, in most studies, the designed guidelines are not considered as clinical and comprehensive. This review was conducted to design and develop an evidence‐based guideline for optimal hand burn care. Yet, the research question is how the nursing care guideline in burned hands is and can its effects improve the performance of burned hands?

## 2 AIMS AND MATERIALS

Clinical guidelines are systematically developed statements that help the healthcare team and their patients to make appropriate decisions about a special condition or treatment. As stated in studies (Butler, Hall, & Copnell, 2016; Gallery, Volunteers, & Login, 2007; Committee, 2018; Organization, 2014), it is necessary to design an integrative review (IR) for developing guidelines. Therefore, we designed an IR for developing a nursing care guideline for patients with burned hands; the IR method is an approach that, by combining different methods and examining all the findings of particular issues or subjects, provides useful and valuable information to the researcher or practitioners on that subject (Whittemore & Knafl, 2005). In this research, we used the Whittemore and Knafl's (2005) IR framework stages, which included Problem identification, Literature search, Data evaluation, Data analysis and Presentation.

Integrative reviews are the broadest type of research review methods permitting for the concurrent inclusion of experimental and non‐experimental research to more fully understand a phenomenon of concern. These reviews include a wide range of purposes: to define concepts, to review theories, to review evidence and to analyse methodological issues of a particular topic (Broome, 2000).

### 2.1 Stage 1: Problem identification

The first step in the review method is a clear identification of the problem; then, variables of interest are defined theoretically and practically. Clinical guidelines are specified and extended guides with different variables in a specific field. According to studies, implementation of guidelines can improve the proficiency of the healthcare team in the management of patients, reduces mortality and morbidity rate and minimizes complications of the inappropriate treatment plans (Brychta, 2012; Gallery et al., 2007; Committee, 2018). Due to the importance of evidence‐based guidelines in managing hand‐burned patients and lack of these guidelines in our context, we aimed to design an IR for developing an evidence‐based guideline for hand burn patients.

### 2.2 Stage 2: Literature search

This research question was designed based on the Setting, Perspective, Intervention, Comparison and Evaluation (SPICE) framework that is more helpful than Population, Intervention, Comparison and Outcomes (PICO) framework with two important changes. These changes included dividing the population element into both “setting” and “perspective” and “evaluation” instead of outcomes (Cleyle & Booth, 2006; Crumley & Koufogiannakis, 2002). These new concepts of the SPICE framework confirm that data practice is a social science and incorporates other concepts such as “outputs” and “impact” together with less tangible effects of a library or instructional intervention (Cleyle & Booth, 2006). SPICE framework is a more appropriate framework for health and social sciences (Cleyle & Booth, 2006; Eldredge, 2001) and helps practitioners to identify their practice‐based questions. This framework was also used for matching the research design to the question, and inclusion and exclusion criteria, and guides the database search strategy (Cleyle & Booth, 2006).

#### 2.2.1 Inclusion and exclusion criteria

Eligible articles for this review include RCT, experimental, semi‐experimental, descriptive and systematic review studies on guideline, protocols or management of adult hand burns, dressing, wound healing, exercise, hand physiotherapy, burn pain and hand burn studies available to the full‐text article in English or Persian language, published from January 2000–August 2019.

Articles were excluded if studies were on animal burns or the study design was in pilot design studies, newsletters and case report.

#### 2.2.2 Designing the search strategy

All databases were searched using the terms: (hand(s) burns OR Forearms burns OR hand(s) Burns wound OR Hand(s) injury OR Injury forearms OR Forearms burns wounds) AND (guidelines OR protocol) AND (Care OR management) AND (Dressing OR Dressing gloves) AND (Nutrition OR Diet) AND (Exercise OR Physiotherapy OR Rehabilitation) AND (Pain) (Pruritus OR Itch) AND (Education).

In this study, eleven appropriate databases were used: EMBASE, PubMed, Web of Science, SCOPUS, Clinical Key, Cochran, CINAHL, Iranmedex, Magiran, Scientific Information Database (SID) and Google Scholar.

To determine the searching method and determine the inclusion and exclusion criteria, a research group formed on 15 February 2018. At first, two researchers (HA and MK) independently searched the articles based on the search terms in the EMBASE, Cochran, CINAHL, PubMed, Web of Science, SCOPUS, Clinical Key, Iranmedex, Magiran, Scientific Information Database (SID) and Google Scholar from January 2000–August 2019 without any language limitations; grey literature search was conducted using professional databases and dissertations (master's and Ph.D.). All records were screened based on title and abstract, and then, the final papers were extracted according to the inclusion and exclusion criteria of the study.

**Figure 1:**
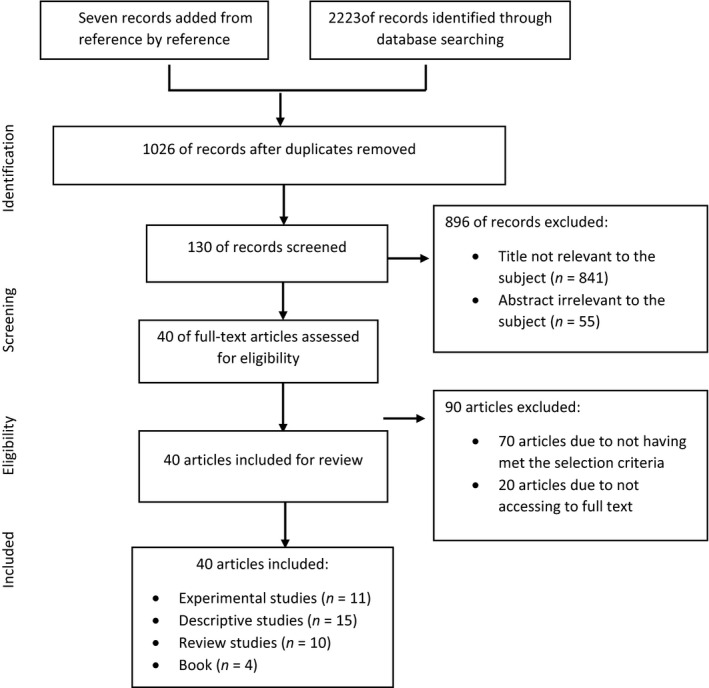
PRISMA flow chart showing article selection stages

The initial search resulted in 2,230 records from databases, grey literature and reference by reference based on the search terms. Subsequently, 1,204 papers were duplicates and excluded from the study, and the total records identified were 1,026. A total of 896 articles were excluded due to irrelevant titles (*N* = 841) and irrelevant abstracts (*N* = 55). The other 130 records were screened for full text. Then, 90 records were excluded because of not meeting inclusion criteria. In addition, twenty of the studies were excluded because we did not have access to the full text. Finally, 40 articles were identified consistent with the inclusion and exclusion criteria. The process of identifying, evaluating and selecting articles is presented based on Preferred Reporting Items for Systematic Reviews and Meta‐analyses (PRISMA; Moher, Liberati, Tetzlaff, & Altman, 2009; Figure 1).

### 2.3 Stage 3: Data evaluation

Mixed‐studies review (MSR) can be more appropriate for decision‐makers and practitioners by providing a rich and practical understanding of complex health interventions and programmes (Pace et al., 2012). The Mixed Methods Appraisal Tool (MMAT) seems to be a useful and unique tool for evaluating MSR (qualitative, quantitative and mixed methods) (Hong, Fàbregues, et al., 2018). This tool is recommended by the National Institute of Excellence in Health Services in Quebec (INESS) and increasingly popular because of their potential for addressing complex interventions and phenomena, specifically for assessing and improving clinical practice (Hong, Gonzalez‐Reyes, & Pluye, 2018).

The MMAT was developed in 2006 and was revised in 2011 and 2018 (Hong, Pluye, et al., 2018; Pace et al., 2012). This appraisal tool can be used to appraise the quality of empirical studies including primary research based on experiment, observation or simulation. Besides, the MMAT permits the appraisal of the most common types of study methodologies and designs (Hong, Pluye, et al., 2018).

The MMAT contains five specific sets of criteria: (a) a “qualitative” set for qualitative studies; (b) a “randomized controlled” set for randomized controlled quantitative studies; (c) a “non‐randomized” set for non‐randomized quantitative studies; (d) an “observational descriptive” set for observational descriptive quantitative studies; and (e) a “mixed‐methods” set for mixed‐methods research studies, with design components of mixed‐methods research. Each study type is reviewed and evaluated in its methodological domain (Pace et al., 2012).

The eligibility of articles was discussed in the research team; then, to appraise the selected papers in this study, two reviewers independently evaluated the quality of the papers with the MMAT method. We use this method to evaluate the quality of selected studies and to increase the validity of the study.

For evaluating the studies at first, we studied all relevant articles to identify their design. Then, we evaluated and scored them based on the MMAT checklist for a different type of studies. For example, we assessed the randomized control studies to see whether they have (a) an “appropriate sequence generation/randomization,” (b) an “allocation concealment and/or blind” and (c) “complete outcome data and low withdrawal.” Finally, we selected the articles with a score of more than 50% for developing our guideline. The articles and scores of two reviewers were examined, and any disagreements were discussed until agreement was reached. According to the evaluation of studies based on MMAT, it was found that the quality of selected studies was moderate with the MMAT score of 50%–75%.

A total of 86 studies were excluded due to not meeting the study's inclusion criteria during the quality appraisal of the articles by the research team. The quality scores, study aim, design, data collection, conclusion and limitation of the studies are included in Table 1.

**Table 1:** Description of the methodological details, strengths and weakness of studies

Author(s)/ year & country	Research aim	Study design	Study population	Data collection	Strengths and Weakness/ limitation	MMAT score (%)
Kamolz et al. (2009) Australia	To introduce principles of hand burn treatment, wound management, surgical treatments, etc…	Review	–	–	Presenting hand burn management for all types of burns (scald, chemical, electrical), including surgical methods for managing deep partial hand burns, offering different methods of wound treatment and wound covering are the key strengths of the study Lack of introducing the review methodology, study population, data collection and setting are the main challenges in this article.	50
Rousseau, Losser, Ichai, and Berger (2013) Switzerland	To provide evidence‐based recommendations for clinical practice	GRADE methodology (Grade of Recommendation, Assessment, Development and Evaluation)	PubMed search including human studies 1979 through 2011	–	Offering a detailed guide for all types of micronutrients necessary for burn patients and classifying the key result of the topic by A, B, C and D groups are two important strengths of the study Unclear review methodology, study population, data collection and setting are the main challenges to this study.	75
Jafari et al. (2018) Switzerland	To analyse losses of 12 TEs and Mg through burn wound exudation and corresponding plasma concentrations during the first week after burn injury and to evaluate the impact of current TE (trace elements) repletion protocols	A prospective observational study without intervention	15 adult patients burned 29 ± 20% of body surface (TBSA)	Checklist	The first study that provides a kinetic view of essential tests (trace elements) in exudates and serum after severe thermal burns	50
Li, Dai, et al. (2017) china	To observe the effect of a rehabilitation intervention on the comprehensive health status of patients with hand burns.	A randomized clinical trial	60 patients with hands burn	The Abbreviated Burn‐Specific Health Scale	As the article states the model was only suitable for hand‐burned patients in hospital and in addition, according to the limited number of studies in burn rehabilitation in China, the exact impact is unknown.	75
Pantet, Stoecklin, Vernay, and Berger (2017) Switzerland	To appraise the impact of the differences in our nutritional practice, general compliance with the guidelines and potential outcomes	A retrospective cohort study	All consecutive burns admissions to the ICU, between 1 June 1999– 31 December 2014	Checklist	Failure in follow‐up discharged patients for evaluating the outcomes.	50
Alsbjörn et al. (2007) Denmark	To improve the overall outcome for community‐treated patients in the expanding European Union and reducing the number of preventable late referrals to specialists	Descriptive	–	–	Devising a new treatment algorithm to provide clear and current guidance on the management of partial‐thickness burns in the general hospital and community setting	50
Richardson and Mustard (2009) UK	To introduce a comprehensive study for the management of pain in burn units	Review	–	–	Discussing both pharmacological and non‐pharmacological methods of burn pain management, burn‐related pain types, introducing standardizations and guidance are the key strengths of the study Unclear review methodology, study population, data collection and setting are the main challenges to this study.	50
Omar and Hassan (2011) Egypt – Saudi Arabia	To compare early excision and skin grafting of burns versus delayed skin grafting in deep hand burns	A randomized clinical trial	40 patients with deep second‐ and third‐degree hand burns	Total active motion (TAM) Hand function using Jebsen–Taylor hand function test (JTHFT)	Evaluating total active motion before and after operating, follow‐up of patients up to 3 months after discharge using Hand function and using Jebsen–Taylor hand function test are study strengths Lack in assessing outcomes such as duration of sepsis, operating hours, wound healing time, skin graft take and long‐term morbidities such as hypertrophic scarring and small sample size (20 patients in each group) are challenges of study.	75
Kwa et al. (2019) Netherlands	To provide a complete overview of all burn debridement technique studied in recent literature and to find the best evidence concerning efficiency and safety	A systematic review	*N* = 27 related studies	–	Presenting overview study characteristics, integrating qualitative and quantities studies results, and complete electronic search strategy	75
Zacharevskij, Baranauskas, Varkalys, Rimdeika, and Kubilius (2018) Lithuania	To compare non‐surgical treatment methods of deep partial‐thickness skin burns of the hand.	Randomized, controlled, parallel‐group, single‐centre clinical trial designed	87 hand‐burned patients	VAS scale (visual analogue scale) Vancouver scale DASH questionnaire	Large sample size, completely presenting the study methodology, good follow‐up programme, and assessing important hand burn‐related outcomes such as pain, hand function, scar formation and healing time are strengths of this study.	75
Afifi et al. (2016) USA‐Egypt	To evaluate the efficacy of skin grafts and flaps in the reconstruction of the postburn hand and wrist deformities	Cross‐sectional descriptive study	57 burn contractures of the wrist and dorsum of the hand	Observational by assessing active range of motion	Small sample size is the major limitation in the article.	50
Robinson and Chhabra (2015) USA	To present hand chemical burn management and describe the management options for chemical burns.	Descriptive	–	–	Unclear methodology, study population, data collection and setting are the main challenges to this study.	50
Summer et al. (2007) USA	To provide an overview of the pain management in burn patients	Critical review	–	–	Providing an overview of the types of pain associated with a burn injury, describing how these different types of pain interfere with the phases of burn recovery and summarizing pharmacologic pain management strategies across the continuum of burn care are study strengths Unclear review methodology, study population, data collection and setting are the main challenges to this study.	50
Arnoldo et al. (2006) USA	In order to review and analyse the available literature in an effort to develop practice guidelines for these two important issues	Summary article	–	–	Unclear review methodology, study population, data collection and setting are the main challenges to this study.	50
Young et al. (2017) USA	In order to review and analyse the available literature in an effort to develop practice guidelines for these two important issues	Summary article	–	–	Unclear review methodology, study population, data collection and setting are the main challenges to this study.	50
Amini (2011) USA	To assess the effectiveness of occupational therapy interventions in rehabilitation of individuals with work‐related forearm, wrist, and hand injuries and illnesses	A systematic review	36 studies commonly in hand rehabilitation	–	Including difficulty representing general conclusions about the results from systematic reviews that were marked by insufficiency of quality studies	50
Sharma and Langer (2019) India	To compare and study the management of hand burns using tangential excision and grafting, and delayed grafting	A randomized clinical trial	84 patients (140 hands)	Questionnaire	Allocating patients into two groups randomly is the more important strengths of study Randomization of subjects into two groups was done keeping in mind the age profile, mode of injury, extent of burns and the time of reporting to this centre are the main challenges in this article.	75
Berger (2009) Switzerland	To discuss the methods of administering nutrition in burn patient	Educational paper (descriptive)	–	–	Presenting the principles of nutritional management of critically ill patients is the study strength.	50
Barillo and Paulsen (2003) USA	To represent hand burn injury management	Review	–	–	Unclear review methodology, study population, data collection and setting are the main challenges to this study.	50
Soni et al. (2017) USA	To present the management of hand burn in the acute phase	Descriptive	–	–	Unclear review methodology, study population, data collection and setting are the main challenges in the study Presenting precise information for common mechanisms of acute hand burns and important aspects of their evaluation and management are the study strengths.	50
Coffey and Thirkannad (2009) USA	To present an easy and inexpensive technique called glove–e‐glove‐gauze method in management of hand burns.	Semi‐experimental	11 hand‐burned patients	Observation	Presenting an easy and inexpensive technique for hand burn dressing is the more significant strength Measuring only active range of motion and did not evaluate other outcomes about pain, pruritus and patients' satisfaction is the challengeable option.	50
Sen, Greenhalgh, and Palmieri (2012) USA	To summarize all the year 2010 burn‐related articles.	Review	More than 1,200 burn‐related articles	–	Grouping articles according to the following: critical care, infection, inhalation injury, epidemiology, psychology, wound characterization and treatment, nutrition and metabolism, pain and itch management, burn reconstruction and rehabilitation categorized.	50
Hsu, Chen, and Hsiep (2016) Taiwan	To investigate the impact of music intervention at dressing change time on burn patients’ pain and anxiety	Prospective, randomized clinical trial	70 burn patients	A numeric rating scale	Small sample size, not double‐blind study and using passive music therapy due to lack of music therapist in their burn centre are the main limitations of the study	75
Brychta (2012) Netherlands	To present minimum level of burn care provision in Europe	Descriptive	–	–	Presenting a precise definition of guidelines and protocols and introducing a multidisciplinary team including physicians, nurses, occupational therapists and physiotherapists in the treatment of burns are very important strengths.	50
WHO (2007) America	To represent hand burn injury management	Descriptive	–	–	Presenting the principles of burn management of critically ill patients is the study strength.	75
Yastı et al. (2015) Turkey	To guide physicians in the treatment of burn victims until they reach an experienced burn centre.	Review	–	–	The study key strengths are that this review was conducted by a multidisciplinary team including general surgeons, paediatric surgeons, aesthetic, plastic and reconstructive surgeons, anaesthesiologists and intensive care physicians Unclear methodology, study population, data collection and setting are the main challenges in the study.	50
McKee (2010) America	To present acute management of burn injuries to the hand and upper extremity	Descriptive	–	–	Unclear methodology, study population, data collection and setting are the main challenges in the study.	50
Natarajan (2019) India	To shift from preventing malnutrition to disease modulation in nutrition support in critically ill patients	Descriptive	–	–	Offering a detailed guide for all types of micronutrients necessary for burn patients and classifying the key result of the topic by A, B, C and D groups are two important strengths of the study Unclear review methodology, study population, data collection and setting are the main challenges to this study	50
Allam et al. (2007) Egypt	In order to compare to different ointments with polyethylene bag in the management of hand burn complications	Prospective comparative randomized clinical study	106 patients with hand burns	Checklist	Powerful methodology (RCT) and large sample size are the strengths.	75
Mohaddes Ardebili et al. (2014) Iran	To examine the effect of educational programme based on exercise therapy on burned hand function.	Experimental with control group	60 with second‐ or third‐degree burn	Measuring hand function based on Jebsen's hand function test	Time limitation, focusing only on physiotherapy education and failure to follow‐up patients after discharge are main limitation of this study.	75
Sterling et al. (2009) Mexico	To represent hand burn injury management	Descriptive	–	–	Unclear methodology, study population, data collection and setting are the main challenges in the study	50
Abu‐Sittah et al. (2011) Lebanon	To present management of thermal injuries to the hands	Review	–	–	Including American Burn Association Burn Treatment Centre referral criteria is the best strength of this study Unclear review methodology, study population, data collection and setting are the main challenges in the study	50
Fakhar, Rafii, and Orak (2013) Iran	To determine the effect of jaw relaxation on pain anxiety related to dressing changes in burn injuries.	Randomized clinical trial with control group	100 patients with burn diagnoses	Questionnaire	Demonstrating a simple and inexpensive method of jaw relaxation to reduce the pain and anxiety related to dressing change, and large sample size are the major strengths of this article As the authors state, the differences between participants in terms of physiological, emotional, psychosocial and cognitive factors and the different attitudes of dressing room nurses towards patients and its effect on the method of dressing change and the resultant level of pain anxiety are the most limitations.	75
Najafi Ghezeljeh, Mohades Ardebili, and Rafii (2017) Iran	To evaluate the effects of massage and music on pain intensity, anxiety intensity and relaxation level in burn patients.	Randomized clinical trial with controlled	240 burned patients	VAS (visual analogue scale).	Large sample size and allocating patients into three different intervention groups are two important strengths in the study Disability to establish a private and quiet environment for the patients, during the intervention at the hospital even after taking all necessary steps and impossibility to blind the subjects to the study process are the study limitations.	75
Guo, Deng, and Yang (2015) china	To assess the effect of virtual reality distraction on pain among patients with a hand burn undergoing a dressing change.	Randomized clinical trial	94 participants	VAS (visual analogue scale)	Large sample size and allocating patients into three different intervention groups are two important strengths in the study.	75
Curtis (2001) USA	To represent hand burn injury management	Descriptive	–	–	Unclear methodology, study population, data collection and setting are the main challenges in the study	50

### 2.4 Data synthesis

The data analysis stage is one of the most difficult aspects and potentially fraught with error. According to the main object of the study, free line‐by‐line coding of the findings from all studies in Persian and English language occurred. Then, the codes were examined and analysed for their meanings, and similar data were reorganized into two categories (emergency and acute phases) based on the guideline aim and best evidences (Herndon, 2012; Paul, Day, & Williams, 2015; Summer, Puntillo, Miaskowski, Green, & Levine, 2007). All data were examined for meaning and content during the coding process. All similar codes were interpreted and compared impartially in each category, specifically looking for similarities and differences, and then in each section, the best evidence was selected. Predetermined and relevant themes of each category were extracted from all selected studies and compiled into a matrix. The steps of data analysis in the study include data reduction, data display, data comparison, conclusion drawing and verification (Whittemore & Knafl, 2005).

### 2.5 Data reduction and display

To manage data for a better understanding and enhance the visualization of patterns and show the relationships between primary data sources, the following data are considered as the initial subgroups: author, country, year, study design, data collection and results (Table 1). The extracted themes are displayed as an algorithm, and emergency and acute phases are described in Tables 5 and 6.

### 2.6 Data comparison

In the third step of data analysis, data were compared with each other to identify the specific patterns of studies and the precise and important themes in them. Data synthesis from the selected studies was coded by highlighting the relevant parts of the text and assigning code words to these areas. All similar codes were interpreted and compared impartially in each category, specifically looking for similarities and differences, and then, in each section, the best evidence was selected. Following this, a constant comparison was used to develop categories by combining codes. Descriptive themes were attached to each category.

## 3 RESULTS

The papers studied were mainly quantity papers, eighteen articles were conducted using the descriptive design, eleven articles used the experimental or quasi‐experimental or RCT research design, and eleven articles were the Review articles.

### 3.1 Stage 4: Presentation

In the final stage of the framework, more precise details of the primary sources and evidence as a logical chain to provide a result consistent with the findings were given to the reader of the review (Whittemore & Knafl, 2005).

#### 3.1.1 Development of hand burns wound care guideline

Based on two valid and comprehensive references (Herndon, 2012; Paul et al., 2015) in burn care field, the descriptive statements/themes were extracted from our IR and categorized in two main phases (emergency and acute phases), and then, bunt hand management steps were classified based on their priority and importance in caring (Alsbjörn et al., 2007; Brychta, 2012; Yastı et al., 2015). Hand burn management steps, study design, number of related studies and most important descriptive themes depending on each section were included in Tables 2 and 3. Generally, the designed guideline was classified as below:

**Table 2:** Key themes of emergency phase management

Burn phase	Sequence	Steps	Study Design/ authors					Results/key themes
			Experimental	Descriptive	Review	Guidelines/Protocols	Book	
Emergency phase (24–48 hr postburn injury)	1st	Initial patient and wound assessment (*N* = 15)	*N* = 2 Allam et al. (2007), Mohaddes Ardebili et al. (2014)	*N* = 7 Barillo and Paulsen (2003), Kamolz et al., 2009; McKee, 2010; Robinson & Chhabra, 2015; Sterling et al., 2009; Summer et al., 2007)	*N* = 1 Yastı et al. (2015)	*N* = 2 Arnoldo et al. (2006), Brychta, (2012), WHO (2007)	*N* = 3 Buttaravoli and Stephen (2010), Herndon (2012), Ledbetter (2010)	Ensure the patient airway, breathing and circulation is secure Physical examination should be implemented during the initial assessment to estimate burn location, determine the depth and mechanism of injury and assess whether or not there is a vascular compromise in the upper extremity Falls are common in electrical injuries; therefore, the patient is assessed for any secondary traumatic injuries.
	2nd	Cooling (*N* = 5)	–	–	*N* = 2 Abu‐Sittah et al. (2011(, Yastı et al. (2015)	*N* = 1 WHO (2007)	*N* = 2 Buttaravoli Ph and Stephen (2010), Ledbetter (2010)	Immediately, after burn injuries keep the hands under running water to prevent more injuries and minimizing pain.
	3rd	Pain control (*N* = 9)	*N* = 3 Fakhar et al. (2013), Mohaddes Ardebili et al. (2014), Najafi Ghezeljeh et al. (2017)	*N* = 1 Summer et al. (2007)	*N* = 2 Abu‐Sittah et al. (2011), Sen et al. (2012)	*N* = 1 Alsbjörn et al. (2007)	*N* = 2 Herndon (2012), Paul et al. (2015)	Burn‐related pain is extremely variable and categorized into three types: procedural pain, background pain and breakthrough pain Pain management therapy at the emergency phase applies only to patients with burn greater than 10% (TBSA). Nevertheless, for patients with extensive hand burn pain, management is necessary Due to potential problems with medication absorption, from the IM and PO route at the emergency phase, the preferred route for the most medications is the intravenous route The visual analogue scale (VSR) has shown to be a reliable method for measuring a patient's pain.
	4th	Wound cleansing (*N* = 8)	–	*N* = 5 Kamolz et al. (2009), McKee (2010), Robinson and Chhabra (2015), Soni et al. (2017), Sterling et al. (2009)	–	*N* = 2 Alsbjörn et al. (2007), WHO (2007)	*N* = 1 Herndon (2012)	It is important to debride any loose or thin blisters and remove any foreign material from the wounds before applying dressings The first step in managing a chemical injury to the hands is decontaminating offending agent Remove all jewellery, rings and watches from burned hand immediately after the burn injury.
	5th	Wound dressing (*N* = 9)	*N* = 1 Mohaddes Ardebili et al. (2014)	*N* = 5 Barillo and Paulsen (2003), Kamolz et al. (2009), McKee (2010), Soni et al. (2017), Sterling et al. (2009)	–	*N* = 1 Alsbjörn et al. (2007)	*N* = 2 Herndon, (2012), Paul et al. (2015)	The main aim of all burn dressings and wound care is to prevent infection and fluid loss, decrease pain, and accelerate wound closure and re‐epithelialization, and it must be simple enough to permit the hand to have a full passive and active range of motion Cover the hand wound with a sterile gauze bandage or a silicon sheet.
	6th	Hand positioning (*N* = 2)	–	*N* = 1 Barillo and Paulsen (2003)	–	*N* = 1 WHO (2007)	–	The most important step in hand management at emergency phase is hand elevation Elevate burnt hands above the level of heart on pillows to improve circulation and minimize oedema.
	7th	Nutritional support (*N* = 5)	–	*N* = 1 Natarajan (2019)	*N* = 2 Jafari et al. (2018), Pantet et al. (2017)	*N* = 2 Rousseau et al. (2013)	–	The main purpose of nutritional support in burn patients includes the following: to accelerate good wound healing to prevent and control infections to prevent protein loss and body mass Initial nutrition assessment should do at admission day for developing baseline data to distinguish the progress made during the therapy The patient feeding should be initiated in the first 24–48 hr of postburn injury, and their diet should include a variety of micro‐ and micronutrients including proteins, vitamins, carbohydrates, fats and minerals.

**Table 3:** Key themes of acute phase management

Burn phases	Consequence	Steps	Study Design/ authors					Results/key themes
			Experimental	Descriptive	Review	Guidelines/protocol	Book	
Acute phase (>48 hr postburn injury till wound closure)	1st	Daily patient and wound assessment (*N* = 5)	–	*N* = 3 Curtis (2001), Richardson and Mustard(2009), Soni et al. (2017)	–	*N* = 1 WHO (2007)	*N* = 1 Paul et al. (2015)	It is not always possible to estimate burn depth at first day of injury, so you may need to assess burn depth for 72 hr postburn Daily physical examination should be performed to assess wound characteristics, infection signs, systematic or local antibiotic requirement, and pain severity, passive and active range of motion, excision and grafting requirement Assess the surrounding tissue for signs of cellulitis.
	2nd	Pain control (*N* = 8)	*N* = 3 Guo et al. (2015), Hsu et al. (2016), Najafi Ghezeljeh et al. (2017)	*N* = 1 Sterling et al. (2009)	*N* = 1 Summer et al. (2007)	*N* = 1 Alsbjörn et al. (2007)	*N* = 2 Herndon, (2012); Paul et al. (2015)	The visual analogue scale (VSR) has been shown to be a reliable method for measuring a patient's pain After the emergency phase has been completed, the patient may be tolerating oral pain medications Use of pain relief medications such as opioid agents, NSAIDs, lidocaine and acetaminophen to control procedural pain according to physician prescription, before and during wound manipulating Massage is considered as an effective method to reduce background and breakthrough pain, due to the prevention of muscle spasm Music can decrease the pain level by reducing sympathetic activities and releasing endorphin.
	3rd	Wound cleansing (*N* = 8	*N* = 2 Omar and Hassan (2011), Sharma and Langer (2019)	*N* = 4 Barillo and Paulsen (2003), Kamolz et al. (2009), McKee (2010), Sterling et al. (2009)	*N* = 1 Kwa et al. (2019)	*N* = 1 Alsbjörn et al. (2007)	–	Burned hands should wash at least once daily with water and mild soap Early excision and grafting increases wound healing with better functional and aesthetic outcomes The physician should choose a best available debridement method for hand burn wound cleansing including conventional tangential excision (CTE), hydro surgery (HS), enzymatic debridement (ED) and shock waves (SW) Moist wound environment promotes autolysis debridement during which burn wounds are naturally cleaned from necrotic tissue.
	4th	Wound dressing (*N* = 14)	*N* = 3 Allam et al. (2007), Coffey and Thirkannad (2009), Zacharevskij et al. (2018)	*N* = 5 Barbosa‐García, (2009), Barillo and Paulsen (2003), McKee (2010), Robinson and Chhabra (2015), Sterling et al. (2009)	*N* = 2 Fortner (2012); Yastı et al. (2015)	*N* = 2 Alsbjörn et al. (2007), WHO (2007)	*N* = 2 Herndon (2012), Paul et al. (2015)	Wound dressing choices depends on several factors, including surgeon priority, wound location, wound bed characteristics and patient age. Use the treatment choices described below, for dressing hand burns based on wound characteristics and surgeon preference: In superficial burns, application of moisturizing ointment is sufficient In partial‐thickness burns, use paraffin‐impregnated gauze, Acticoat or Aquacel Ag gloves, antimicrobial agents (silver sulphadiazine, mafenide acetate, mupirocin, etc.), and polyurethane film sheet based In full‐thickness burns, use Acticoat or Aquacel Ag gloves, antimicrobial agents and polyurethane film sheet based, also this type of burns may be referred to as surgical (excision or grafting) intervention Keep hands dressing as thin as possible to allow the patient to have early rehabilitation programmes Hydrocolloid dressings promote autolytic debridement by maintaining moist wound environment, and this dressing method minimizes all types of pain and fastest wound healing time, increases epithelialization rate during treatment and improves hand functions.
	5th	Physiotherapy and patient education (*N* = 8)	*N* = 2 Li, Dai, et al. (2017), Mohaddes Ardebili et al. (2014)	*N* = 2 Barillo and Paulsen (2003), Kamolz et al. (2009)	*N* = 1 Amini (2011)	*N* = 1 WHO (2007)	*N* = 2 Herndon (2012), Ledbetter (2010)	Exercise improves circulation, reduces oedema, maintains strength and functional movement and prevents scar contracture Hand rehabilitation is an essential principle in effective care of hand‐burned patients; therefore, the most important step in hand physical therapy is to have a proper educational programme that is easy to understand for the patients It is better to start a hand exercise programme in the first 72 hr after burn injury Physiotherapy programmes should be held, based on patients’ educational needs and hand burn severity in 2 or 3 individual or group educational sessions.
	6th	Nutritional support (*N* = 6)	–	*N* = 1 Natarajan (2019)	*N* = 2 Jafari et al. (2018), Pantet et al. (2017)	*N* = 3 Berger (2009). Rousseau et al. (2013); Young et al. (2017)	–	If it is necessary, it should be coordinated by a nutritionist to evaluate the patient national requirements during the treatment period.

##### Emergency phase

The emergency phase was also referred to as resuscitative phase, which begins with the onset of burn injury and may be completely bypassed in the first 24–48 hr postburn injury. The most important themes that are noticed at this phase are initial patient and wound assessment, cooling, pain control, wound cleansing, wound dressing, physiotherapy and nutritional support.

The key themes included in the emergency phase are as follows: assessment of patients ABC (Soni et al., 2017), assessment of patient for any secondary traumatic injuries (Barillo & Paulsen, 2003), keeping hands under cool water for minimizing deeper injuries (Abu‐Sittah et al., 2011) and removing all foreign bodies from the wound (Robinson & Chhabra, 2015). It is important to debride any loose or thin blisters and remove any foreign material from the wounds before applying dressings (Alsbjörn et al., 2007; McKee, 2010; Soni et al., 2017), covering the hand wound with sterile gauze and bandages(McKee, 2010) and elevating hands for first 48 hr (Barillo & Paulsen, 2003; Paul et al., 2015).

##### Acute phase

The acute phase starts as soon as the emergency phase completely bypassed, and it will continue until wound closure. Duration of this phase may take 2 weeks or more. The most important themes that are noticed in the guideline are daily patient and wound assessment, pain control, wound cleansing, wound dressing, physiotherapy and nutritional support.

The key themes included in the acute phase are as follows: daily assessment of the patient (Curtis, 2001; Herndon, 2012; Kamolz et al., 2009; Paul et al., 2015), physical examination that should be implemented during the daily assessment (Arnoldo, Klein, & Gibran, 2006; Paul et al., 2015), use of pain relievers to control burn‐related pain as physician description (Sterling, Gibran, & Klein, 2009), use of dressing choices as wound bed characteristics and keeping it as thin as possible (Barillo & Paulsen, 2003), early excision and grafting that increases wound healing with better functional and aesthetic outcomes (Alsbjörn et al., 2007; Omar & Hassan, 2011) and hand rehabilitation, which is an essential principle ineffective care of hand‐burned patients (Amini, 2011; Mohaddes Ardebili et al., 2014). If it is necessary, it should be coordinated by a nutritionist to evaluate the patient national requirements during the treatment period (Berger, 2009; Jafari et al., 2018).

Among 40 articles identified in this review, 23 (52%) focused on the emergency phase, 30 (68%) focused on the acute phase, and 15 (34%) focused on both emergency and acute phases. We have presented a summary of our guideline as an algorithm (Tables 4, 5, 6, 7, 8). Knowing that, five of the studies were in Persian language and 35 in English language.

**Table 4:**
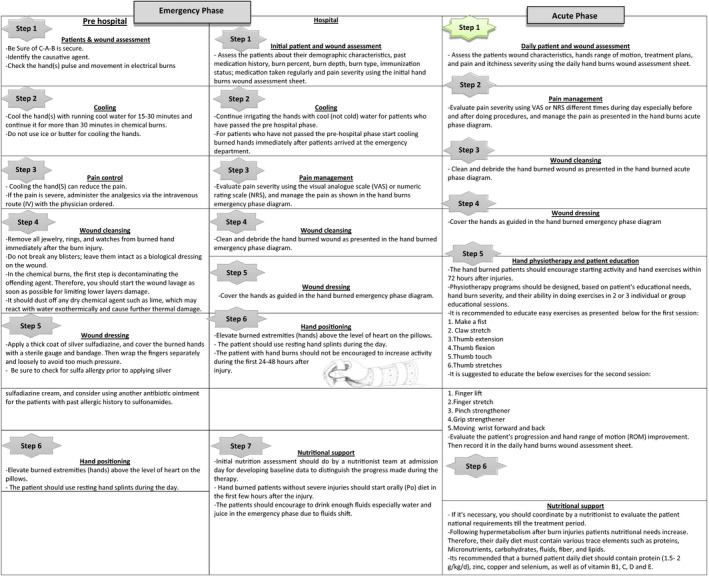
Hand burn management algorithm

**Table 5:** Hand burn emergency phase cleansing, dressing and pain management

Hand burn emergency phase								
Wound degree	Wound cleansing		Dressing		Pain control			
	Hydrotherapy	Wound cleansing	Wounds in proliferation phase with the granulated or epithelize tissue	Wounds in proliferative phase with infected and moderate or high exudate	Pain severity	Background pain	Procedural pain	Breakthrough pain
First	Immediately, after burn injuries keep the hands under running water to prevent more injuries and minimizing pain.	—	Aloe vera gel Vaseline Polyethylene (PE) sheets Olive oil	—	Mild pain (0–3)	Cooling the wound Use Oral analgesics wound moisturizers (vaseline and aloe vera)	Use Oral analgesics Wound moisturizers (vaseline and aloe vera	Adequate management of analgesics and decreasing the interval time of analgesic usage
Second	Immediately, after burn injuries keep the hands under running water to prevent more injuries and minimize pain Continue hand lavage for more than 30 min in chemical burns In solid chemical burns, such as lime, first dust off the agent and then start to lavage Use polyethylene glycol in the burns with phenol like agents.	In the second‐degree burns with blisters, it is suggested: Don't break blisters with less than 2cm diameter except those are on the joints Aspirate the blisters with more than 2cm diameter	Aloe vera gel Vaseline Polyethylene (PE) sheets Olive oil	Daily dressing in 1% silver sulphadiazine or mafenide ointment and vaseline for wounds with high amount of exudate Daily dressing with 2% mupirocin or bacitracin and vaseline for wounds with low amount of exudate Dressing in silver aqua gloves and changing herring 14–7 days Daily dressing with antibiotic ointment and polyethylene gloves	Mild pain (0–3)	Use analgesics (oral, rectal) before procedures	Use analgesics (oral, suppository) before procedures	Adequate management of analgesics and decreasing the interval time of analgesic usage
					Mode pain (4–6)	Use analgesics (oral, rectal) before procedures music therapy, massage therapy before procedures Using Aquacel and polyethylene gloves	Analgesic use (oral, rectal, intravenous) with music therapy, massage therapy and jaw relaxation before procedures	Adequate management of analgesics and decreasing the interval time of analgesic usage
					Severe pain (7–9)	Use analgesics (oral, rectal) before procedures music therapy, massage therapy before procedures Using Aquacel and Polyethylene gloves	Analgesic use (oral, rectal intravenous) Use 2% lidocaine ointment on the wound	Adequate management of analgesics and decreasing the interval time of analgesic usage
Third	Immediately, after burn injuries keep the hands under running water to prevent more injuries and minimize pain Continue hand lavage for more than 30 min in chemical burns In solid chemical burns, such as lime, first dust off the agent and then start to lavage Use polyethylene glycol in the burns with phenol like agents	—	Use 1% silver sulphadiazine ointment or 2% mupirocin	Daily dressing in 1% silver sulphadiazine or mafenide ointment and vaseline for wounds with high amount of exudate Daily dressing with 2% mupirocin or bacitracin and vaseline for wounds with low amount of exudate Dressing in silver aqua gloves and changing herring 14–7 days ‐ Daily dressing with antibiotic ointment and polyethylene gloves		Use analgesics (oral, rectal) before procedures music therapy, massage therapy before procedures Using Aquacel and Polyethylene gloves Use 2% lidocaine ointment on the wound	Analgesic use (oral, rectal intravenous) Use 2% lidocaine ointment on the wound	Adequate management of analgesics and decreasing the interval time of analgesic usage

**Table 6:**
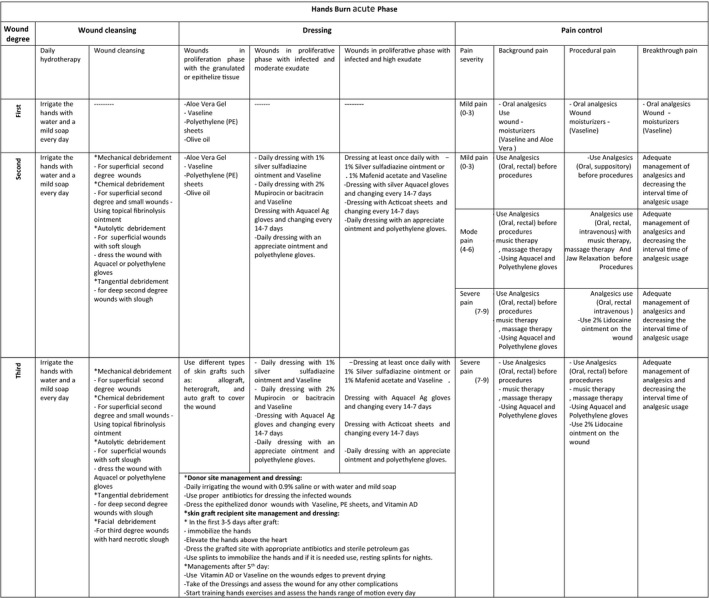
Hand burn acute phase cleansing, dressing and pain management

**Table 7:**
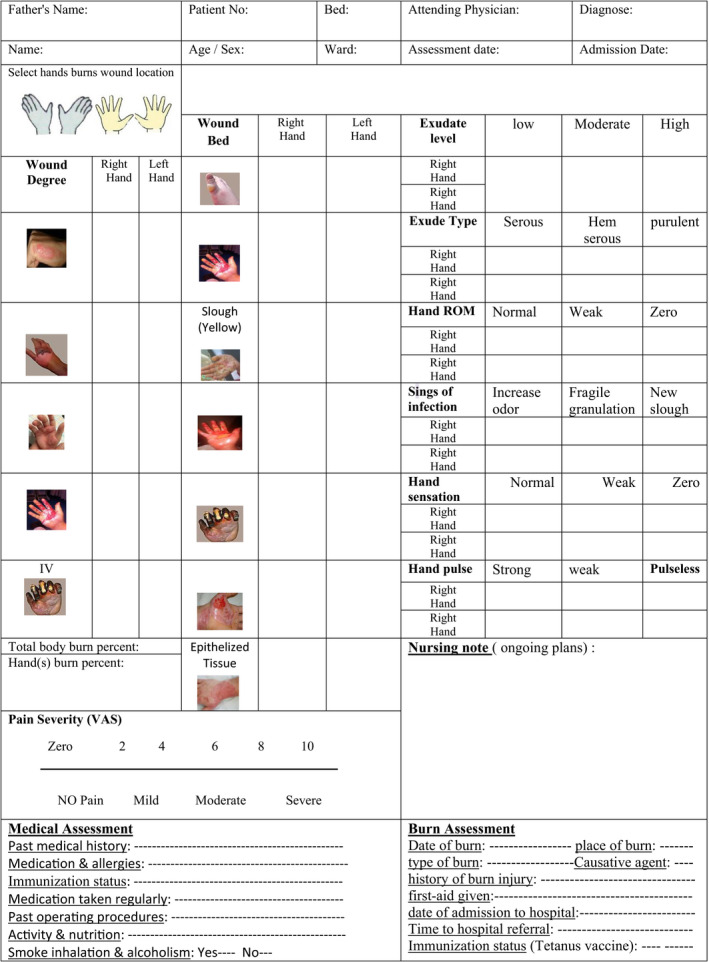
Initial hand burn wound assessment sheet

**Table 8:**
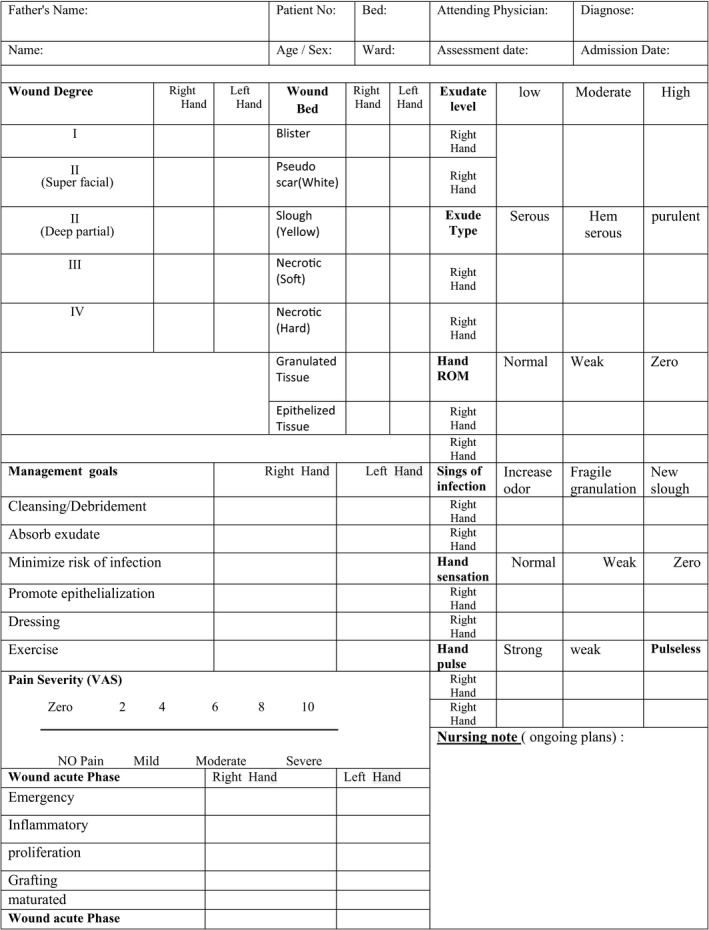
Daily hand burn wound assessment sheet

## 4 DISCUSSION

The latest studies indicate that using evidence‐based guidelines by the healthcare providers is a useful way for presenting proper management for patients with hand burns; therefore, this review aimed to design an integrated and evidence‐based guideline for hand burn management (Esmailian & Golestani, 2016).

According to the author's extensive systematic review, this study has some unique strength, because it provides comprehensive nursing care of hand burns and is designed by qualitative and quantitative study integration. Qualitative studies have been used to extract basic concepts; however, quantitative studies have been used to extract the best management methods. In the study published by the ISBI Practice Guidelines Committee (Committee, 2018), universal recommendations are presented about first‐aid, topical agents in the burn, infection control in the burn, mobility and electrical and chemical burns; nevertheless, it is not specially mentioned hand burn management recommendations.

In this review, we developed a comprehensive and integrated guideline that offers the management of burned hands consecutive in all aspects of caring such as initial and daily assessment, pain control, wound cleansing and dressing, hand physiotherapy and nutritional therapy; however, previous equivalent studies have only provided hands management for specific domains. In review, literature by Lars Peter Kamolz provided only a brief explanation of mechanisms of the injury, escharotomy, treatment of oedema, splinting, wound management, surgical treatment, dressing methods and reconstructions. In the other review study by Abu Sittah (Abu‐Sittah et al., 2011), the management of hands has been only presented for thermal injuries. In another study conducted by Robinson (Robinson & Chhabra, 2015), hand chemical injuries and their management are just discussed. It should be noticed that we have been provided nursing care to all types of burns about chemical, electrical, scald or thermal burns in the guideline.

There are few studies that have considered nursing care for patients with hand burns. In a study of Ashwin Sony (Soni et al., 2017), exhaustive management of hand burns has provided an initial evaluation of the patient, escharotomy, excision, grafting of hand burns, wound management and amputation, but less attention has been paid to nursing care in hand‐burned patients. In the presented study, we aimed to show the importance of nursing care in the management of hand‐burned patients; moreover, in the guideline, a multidisciplinary team consists of a physician, nurses, nutritionist and physiotherapist considered for hand burn management. In addition, we provided the nursing cares in two phases, respectively, according to their importance in the management of hand‐burned patients: in the emergency phase, initial patient and wound assessment, cooling, pain control, cleansing, dressing, hand positioning, and nutritional support are presented; and in the acute phase, daily wound and patient assessment, pain control, wound cleansing, wound dressing, hand physiotherapy and nutritional support are presented.

Accordingly, it is clear that our systematic review results are largely in line with other studies (Arnoldo et al., 2006; Young et al., 2017) that show implementation of evidence‐based guidelines increases the quality of nursing care, reduces burn injury complications, reduces the distance between the theoretical and clinical aspects and helps the decision‐making of the multidisciplinary burn teams and patients in specific clinical conditions. In this interest, the results in a retrospective cohort study conducted by Clark, Lowman, Griffin, Matthews, and Reiff (2013) indicate that implementation of early mobilization guideline on burn patients collaborates the multidisciplinary team (physician, nurses, physiotherapist) in early patients’ mobility and reduces burn injury complications such as airway, cardiovascular, gastrointestinal, musculoskeletal and deep vein thrombosis, while no adverse events were reported related to the early mobility guideline. In another study by Ratcliff, Stephen (English, Ratcliffe, & Williams, 1999) with the aim of evaluating the effect of pain and anxiety guideline in children with burns shows that implementation of pain control guidelines such as music therapy guidelines is more effective than routine pain management on procedural pain control in burn patients.

The other advantage of this study is that all types of study including randomized control trials, descriptive studies, reviews and books, selected in the systematic review, were used for the development of nursing care guidelines after their quality was evaluated by the MMAT method. So studies with unclear methodology and results, case reports and pilot studies were not used in designing the guideline.

In conclusion, the unique property of this study is that it is evidence‐based. In other words, a strong systematic review was conducted to design a guideline that presents all aspects of management for patients with hand burns.

### 4.1 Limitation

This study has some limitations that should be taken into consideration when analysing the results. One of the limitations of this study is that on the one hand, some of the selected quantitative studies did not include enough population to do generalization and that on the other hand, some of the qualitative studies were originated from very different contexts also that we did not have access to the full text of all selected studies. In addition, the inability to use original language studies is another limitation of our study.

## CONFLICT OF INTEREST

The authors declare that they have no conflicts of interest.

## ETHICAL APPROVAL

The ethics committee of Tabriz University of Medical Sciences authorized the permission to conduct this study (Ethical No: IR.TBZMED.REC.1396.975). All authors have full control of all primary data, and they agree to allow the journal to review their data if requested.
